# Communications

**Published:** 1867-05

**Authors:** 


J. A. McCLELLAND.

---

LOUISVILLE, *Dec.* 22*d*, 1866.

DR. J. A. McCLELLAND,

*Dear Sir:*—I have made a chemical examination of the "Sozodont," (liquid and powder) which you left with me. I find the liquid contains Alcohol, Water, Soap, Essential Oil, Sugar, and a little extractive matter.

The Powder contains Starch, Gypsum (Plaster of Paris), and Powd. Orris Root.

Yours very Respectfully,

THOS. E. JENKINS, M. D., *Chemist.*

